# Phenological drivers of ungulate migration in South America: characterizing the movement and seasonal habitat use of guanacos

**DOI:** 10.1186/s40462-022-00332-7

**Published:** 2022-08-13

**Authors:** Malena Candino, Emiliano Donadio, Jonathan N. Pauli

**Affiliations:** 1 Department of Forest and Wildlife Ecology, University of Wisconsin, Madison, WI 53706 USA; 2 Fundación Rewilding Argentina, CP 1425 Buenos Aires, Argentina

**Keywords:** Phenology, Plasticity, Green waves, Snow cover, *Lama guanicoe*, GPS data

## Abstract

**Background:**

Migration is a widespread strategy among ungulates to cope with seasonality. Phenology, especially in seasonally snow-covered landscapes featuring “white waves” of snow accumulation and “green waves” of plant green-up, is a phenomenon that many migratory ungulates navigate. Guanacos (*Lama guanicoe*) are native camelids to South America and might be the last ungulate in South America that migrates. However, a detailed description of guanacos´ migratory attributes, including whether they surf or jump phenological waves is lacking.

**Methods:**

We quantified the migratory movements of 21 adult guanacos over three years in Patagonia, Argentina. We analyzed annual movement patterns using net squared displacement (NSD) and home range overlap and quantified snow and vegetation phenology via remotely sensed products.

**Results:**

We found that 74% of the individual guanacos exhibited altitudinal migrations. For migratory guanacos, we observed fidelity of migratory ranges and residence time, but flexibility around migration propensity, timing, and duration of migration. The scarce vegetation and arid conditions within our study area seemed to prevent guanacos from surfing green waves; instead, guanacos appeared to avoid white waves.

**Conclusion:**

Our study shows that guanaco elevational migration is driven by a combination of vegetation availability and snow cover, reveals behavioral plasticity of their migration, and highlights the importance of snow phenology as a driver of ungulate migrations.

**Supplementary Information:**

The online version contains supplementary material available at 10.1186/s40462-022-00332-7.

## Introduction

Seasonality, the temporal periodic fluctuations in climatic conditions, plays a major role in how animals interact with their environment [1]. In general, seasonal habitats consist of alternating favorable and unfavorable periods; winter being a particularly challenging period characterized by low temperatures, snow cover, resource limitation and energetic deficits [2, 3]. In response to the constraints of winter, many species have evolved strategies to avoid winter conditions via seasonal migration [4]. Migration encompasses a variety of periodical round trip movements between discrete, non-overlapping home ranges [5] and arises in systems where seasonality is predictable [6] and the benefits of alternating between ranges exceeds the energetic costs and risks associated with long distance movement [7, 8].

Among ungulates, migration is a particularly important strategy to track food availability and reduce competition and predation [9, 10]. While some species have high fidelity in their migration habits [11, 12] including well-defined corridors, ranges that are regularly returned to and relatively fixed departure dates, other species exhibit high levels of plasticity in their migratory behavior. These differences occur even within populations [7, 13]. Some species for example, are partially migratory [14], where some individuals remain resident while others migrate, or where the same individual can alternate between these two strategies. Although originally understood as a particularity of some species, partial migration is now seen as the rule among ungulates [15]. Partial migration is often attributed to extrinsic factors such as precipitation and resource availability, and demographic factors such as animal density and individual age [16]. This strategy appears to be beneficial when facing unpredictable seasonality and heterogeneous, complex landscapes [17].

There is growing recognition around plasticity on other migration characteristics (e.g., distance, timing, and duration) as a strategy to deal with a changing environment [15]. Migration distances range from < 15 km [18], especially common in mountainous species that exhibit short altitudinal movements [7, 8, 19], to > 1600 km, in species that inhabit low elevation plains and follow precipitation gradients and high quality vegetation [9, 20, 21]. Timing and duration in migration are also flexible as ungulates respond to interannual and spatial variation in green-up, snow melt and rainfall [9, 10, 22]. Indeed, tracking vegetation green up to maximize intake of high quality forage, or “surfing the green wave” [23], is recognized as the predominant driver behind ungulate migrations [24, 25]. Other species, however, jump green waves by accelerating their migration to arrive at summer ranges before vegetation greenness peaks, which could be a product of non-suitable habitat along the way or due to short migrations that do not allow surfing [26].

While the phenology of vegetation is a recognized driver of ungulate migrations, the importance of snow is less understood. However, several studies showed that snow presence and snow characteristics influence migration parameters. Severe winter weather and snow cover and depth have been found to be related to the onset of both fall and spring migrations [27, 28]. The timing of fall departure can be influenced by the trade-off between extending access to summer range foraging grounds and the risk of encountering deeper snow along the way which leads to higher energy demands related to locomotion as well as the struggles of accessing snow covered vegetation [29]. Spring migration can also be affected by snow depth and snow melt dates [27], which is unsurprising given the correlation of snow presence and vegetation productivity, particularly in dry landscapes where precipitation occurs mainly as snow [30]. Species that inhabit more extreme latitudes that feature longer snow seasons, typically spend more time in their winter than summer ranges and exhibit delayed migration starting dates compared to southern species [31].

The guanaco (*Lama guanicoe*) is a camelid native to South America [32]. A monomorphic and social species, the guanaco inhabits a wide range of habitat types [33]. Guanacos are the only known South American ungulate with migratory populations [34, 35]. However, data on guanaco migration movements is scanty and based on seasonal changes in guanaco abundances and radiotracking of a few individuals. Thus, a detailed description of the attributes of guanaco migration is lacking. Moreover, whether migration is a plastic trait among guanacos is unknown because previous studies last ≤ 1 year. Further, the drivers of guanaco migration are also unknown, and it is unclear whether guanacos surf or jump green waves or respond to other climatic factors or resource pulses.

To describe the movement strategies and understand the factors driving guanaco migration we studied a wild guanaco population during three migratory cycles in southern South America. We examined the spatial and temporal features that characterize this behavior, as well as their variability. To understand their seasonal habitat use, we analyzed location and size of their summer and winter home ranges, as well as their movement dynamics within them. Given the elevational gradient and seasonality characteristic of our study area, we hypothesized that guanacos would be migratory and generally track favorable seasonal conditions of plant availability and snow presence. More specifically, due to the topological and phenological complexity of the area and lack of competition with domestic cattle, we predicted the population to be partially migratory along an altitudinal gradient, with summer home ranges at higher altitudes than winter ones. Given the reduced productivity during winter as well as larger social groups during this season [35], we expected guanacos to move more during this season while foraging, leading to larger winter home ranges compared to summer ones. Finally, given the scarce vegetation typical of the study area, which is composed of a combination of grasses that show vegetative growth during winter and early spring, and shrubs that concentrate phenological activity during the summer [36], we predicted that green-up phenology would not be a smooth wave-like progression and prevent green-wave surfing. Consequently, we predicted that the phenology of snow cover, would become more relevant in mediating migratory characteristics.

## Materials and methods

### Study area

**Fig. 1:**
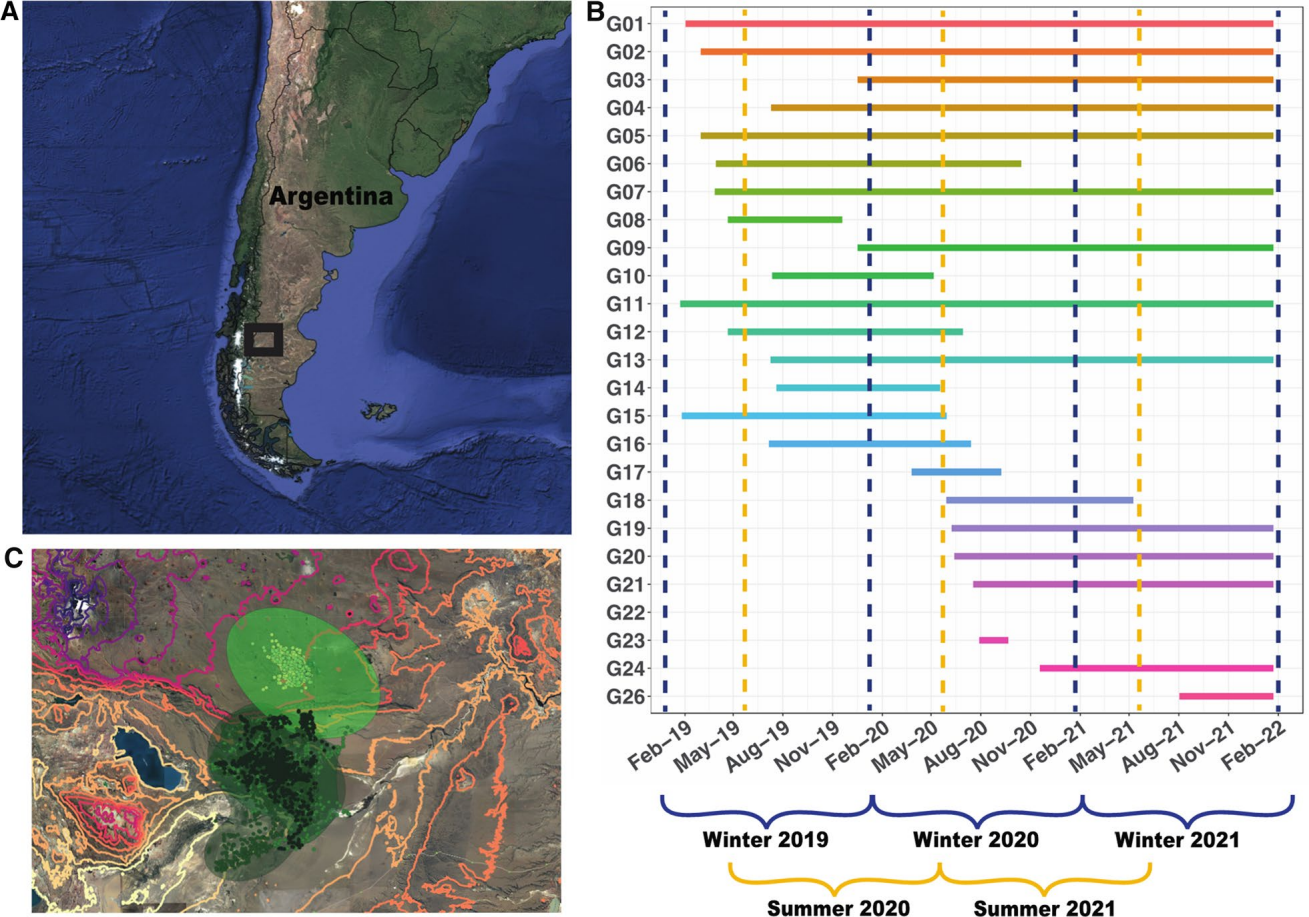
**A** Study Area in Patagonia Argentina, where 25 adult guanacos (*Lama guanicoe*) were darted and fitted with GPS-Iridium collars. **B** Timelines for individual guanacos illustrating capture and death or end of study dates. Dark blue dotted lines indicate migration analysis starting date for individuals that were captured in their summer ranges and experienced a winter migration. Yellow dotted lines mark starting date for individuals that were captured in their winter range and experienced a summer migration. Individuals that were not collared long enough to complete a migration period (G08, G17, G22, G23) were excluded from the analysis. **C** Altitudinal contour curves (lower elevations represented by lighter colors), GPS locations and 95% seasonal Autocorrelated Kernel Density Estimate (AKDE) home ranges with color gradients for one migratory guanaco (G02) illustrating summer (light green) and winter (dark green) ranges

The study area was located in the Patagonia region of Argentina; it encompassed 131,100 ha and included part of the recently created Patagonia National Park and private lands (Fig. 1A). The climate is cold with mean temperatures of 5 °C, and strong winds that prevail during the summer. Precipitation ranges from 100 to 250 mm falling mainly in winter and spring primarily as snow, and in the form of dew that occur year-round [37]. The landscape is a grass-dominated steppe with high elevated plateaus that create a west-east steep elevational gradient. To the north-west the area is characterized by higher altitudes represented by the Buenos Aires plateau (1200–1600 m.), where most of our captures occurred. These plateaus are intersected by several tributaries of the Ecker River and have numerous permanent and ephemeral ponds. To the south-east we find lower altitude areas (200–600 m.) intersected by canyons with ephemeral vegas. The vegetation throughout the study area rarely exceeds 0.5 m in height [38] and is composed mainly by graminoids (e.g., *Stipa* spp., *Festuca* spp., *Poa ligularis*) and shrubs (e.g., *Berberis heterophylla*, *Junelia tridens*).

### Animal capture and data collection

From January 2019 through February 2021, we darted 25 free-ranging guanacos (9 females and 16 males) and fit them with GPS collars (LiteTrack Iridium 420 collar; weight: 0.6 kg; Lotek, Ontario, Canada). Only one adult guanaco per social group was collared. We used a CO_2_ rifle and 1.5 ml darts, with a dose of 5 mg (0.05 mg/kg dose) of fentanyl oxalate (Thianil, Wildlife Pharmaceuticals) as an immobilization agent, and a dose of 10 mg of naltrexone hydrochloride (Trexonil, Wildlife Pharmaceuticals) for every mg of Thianil as an antagonist agent. All applicable institutional and/or national guidelines for the care and use of animals were followed (IACUC protocol ID: A006515). We programmed the GPS collars to collect a fix every two hours. Four individuals died before completing a migration period and were excluded from the analysis. For the remaining individuals we eliminated locations that had a dilution of precision (DOP) > 10 (1.6%) and locations with altitudes that were unfeasible (2.6%). Data management and statistical analysis were conducted using R [39]. Fieldwork and captures were performed under permits from Santa Cruz province’s Wildlife Agency and subsequent renewals (Disp #19, Exp #492749/18).

### Migration classification and parameters

To establish the occurrence of migration we combined two different methods. First, we used the Net Squared Displacement (NSD) analysis which measures the distances between the starting location and the subsequent relocations for the movement path of a given individual, and then fits the data using a priori models representing migratory, disperser and resident behaviors [31, 40, 41]. We randomly selected one location a day to thin the data in order to improve model convergence [40, 41], and visually inspected each individual’s relocations to determine the most appropriate starting season and date for each migratory cycle (Fig. 1B). We then fit NSD a priori models and classified individuals to migratory, resident or disperser strategies based on model ranking (Akaike’s information criterion [AICc]) (Additional file 1: Figs. S1 and S2). For migratory animals, we determined the timing, duration and distance parameters using the package *MigrateR* [41]. We estimated the altitudinal differences between seasonal ranges (*δ*), which corresponds to the asymptote of the double sigmoidal curve from the migratory NSD model for each guanaco. We also obtained temporal parameters of migration timing (*θ*; the midpoint of the departing movement represented by the inflection point of the curve), migration duration (*ρ*; the period of time the individual occupied each seasonal range), and the duration of the migratory movement *per se* (*φ*; the time required to complete ½ to ¾ of the migration). We originally fit both the standard NSD method (i.e., distance) and elevation. We used elevation obtained from our GPS collars after corroborating accuracy by testing for correlation with altitudinal data from the Terrain Elevation Data 2010 (GMTED2010) (R = 0.99). When comparing methods we found that while both models yielded similar results (Additional file 1: Table S1), the elevation model was a better fit our data (Additional file 1: Fig. S3). Consequently, we report NSD results for the analysis of elevation (Additional file 2).

For our second method, we estimated overlap of seasonal ranges [42]. We first classified each guanaco’s location to its summer range, winter range or to periods of active migration, based on the migration timing parameters from the NSD analysis, using mean migration date values obtained from guanacos classified as migratory, for those individuals classified as residents or dispersers by NSD that did not have estimated parameters. We calculated the 95% Kernel Density Estimate (KDE) for each seasonal range in the package *adehabitat* (Fig. 1C) [43] and evaluated the Bhattacharyya’s affinity index overlap (BA) of each individual’s ranges [42, 44]. BA is a measure of affinity between two spatial distributions, ranging from 0 (no overlap) to 1 (complete overlap). We selected overlap thresholds following Cagnacci et al. [42], such that if an individual had two consecutive seasonal ranges (summer-winter) with a BA > 0.15, we classified it as resident for the corresponding period. Furthermore, if alternating seasons had a BA < 0.15 but the overlap between subsequent same season ranges (summer-summer/winter-winter) was < 0.5 we classified that individual as disperser. Otherwise (alternating seasons BA ≤ 0.15, same season BA ≥ 0.5) the individual was considered migratory for that year (Additional file 1: Figs. S4 and S5). If a guanaco was classified as resident or disperser by overlap but as migratory by NSD, we constrained migration distance parameter in NSD to only consider an individual as migratory if its summer and winter ranges had an altitudinal difference > 221.5 m (altitudinal distance [*δ*] 1st quartile for the overall population). If an individual was reclassified as resident when applying this constraint, it was assigned as such.

To confirm that collared guanacos had home ranges for each season as well as to inspect movements within their migratory ranges, we evaluated individual semi-variograms for distances between locations as a function of time. We then estimated seasonal home ranges using the 95% Autocorrelated Kernel Density Estimate (AKDE) estimator as well as home range fidelity, based on the overlap of seasonal AKDE ranges, and average speed of movement within each range in the package *ctmm* [45]. To determine how space use varied throughout the year we tested how home range size was influenced by season (summer, winter) with a generalized linear mixed model in the *lme4* package, where individual guanaco and year were random intercepts [46]. We log transformed our data to assure residuals normality and homoscedasticity and verified model assumptions using the *DHARMA* package. After testing for differences between sexes and finding no significance, individuals were pooled regardless of sex for all analyses.

### Environmental drivers of migration– vegetation green-up and snow

Using Google Earth Engine, we created time series of 16-day composite measurements of the normalized difference vegetation index (NDVI) and the normalized difference snow index (NDSI) for the study area. We used a combination of Landsat and Sentinel 2 satellite imagery at 30 m resolution. If Sentinel and Landsat had data corresponding to the same day, we selected the highest value for the composite; if data from multiple days was available for a particular composite, we used the average. We floored values for both indices at 0 and replaced missing values using temporal linear interpolation. We only used pixels with a quality assessment (QA) bit = 0, and used a threshold of ≥ 10 to determine snow presence [47]. We then created altitudinal ranges of 200 m covering the elevation gradient occupied by guanacos and obtained NDVI and NDSI curves spanning the duration of our study (summer 2019–summer 2022) to visually inspect vegetation and snow cover trends and correlation. We corroborated the association between NDVI values and forage availability by overlaying a landcover map from the European Space Agency (ESA) based on Sentinel-1 and Sentinel-2 data at 10 m resolution, with a mean summer NDVI map based on our NDVI composite, end extracted NDVI values corresponding to different cover types. As expected, NDVI was highest in wetlands, followed by grasslands and shrublands, and was lowest in unvegetated areas (bare soil/sparce vegetation) (Additional file 1: Fig. S6).

Using the same NDVI composites, we obtained measurements for one randomly selected relocation a day for each year of data, and created a space-time-time matrix for each individual guanaco year [26] (Additional file 1: Fig. S7), in which each row represented a spatial location the individual occupied at a certain time (day), and each column was the NDVI value corresponding to that location for every day during the period being analyzed. We calculated the cumulative NDVI value for each location, averaging values by total amount of days to account for differences in individual-year duration. We clustered locations into summer and winter seasonal ranges, and summer and winter migrations according to NSD timing parameters. We calculated the mean NDVI value for each seasonal range to determine what the guanaco would have experienced in terms of vegetation biomass if it would have remained resident in either one of those ranges. Finally, we calculated the values each individual actually experienced by adding up the diagonal values of the matrix and compared the experienced NDVI values with the summer and winter range ones using a generalized linear mixed model analysis with individual guanaco as a random effect. We repeated this procedure with snow cover presence data after using our threshold value to transform the NDSI into a binary index where NDSI < 10 = 0 designated lack of snow cover and NDSI ≥ 10 = 1 indicated presence of snow cover.

## Results

### Migration classification and parameters

We analyzed data from 21 individuals (8 females, 13 males), 9 of which had two or more years of data that we analyzed independently to account for the possibility of an individual switching between strategies (Additional file 1: Table S1), totaling 35 individual years. We classified a total of 26 years, consisting of 12 males and 6 females, as migratory (74.3%). Eighteen of those years were identified as migratory by both methods, whereas 8 were classified as migratory by the NSD and as either resident or disperser by overlap but were ultimately classified as migratory. Nine years (25.7%), 3 males and 4 females, were classified as resident. Three of those were originally classified as non-migratory by both methods while the other 6 were classified as migratory by the NSD and were assigned to be resident after further inspection. Based on their final classification for each year, 5 of the 9 individuals tracked for two years (4 migrants and 1 resident) maintained their strategies throughout the duration of this study, while the other 4 alternated between migratory and resident strategies.

**Fig. 2:**
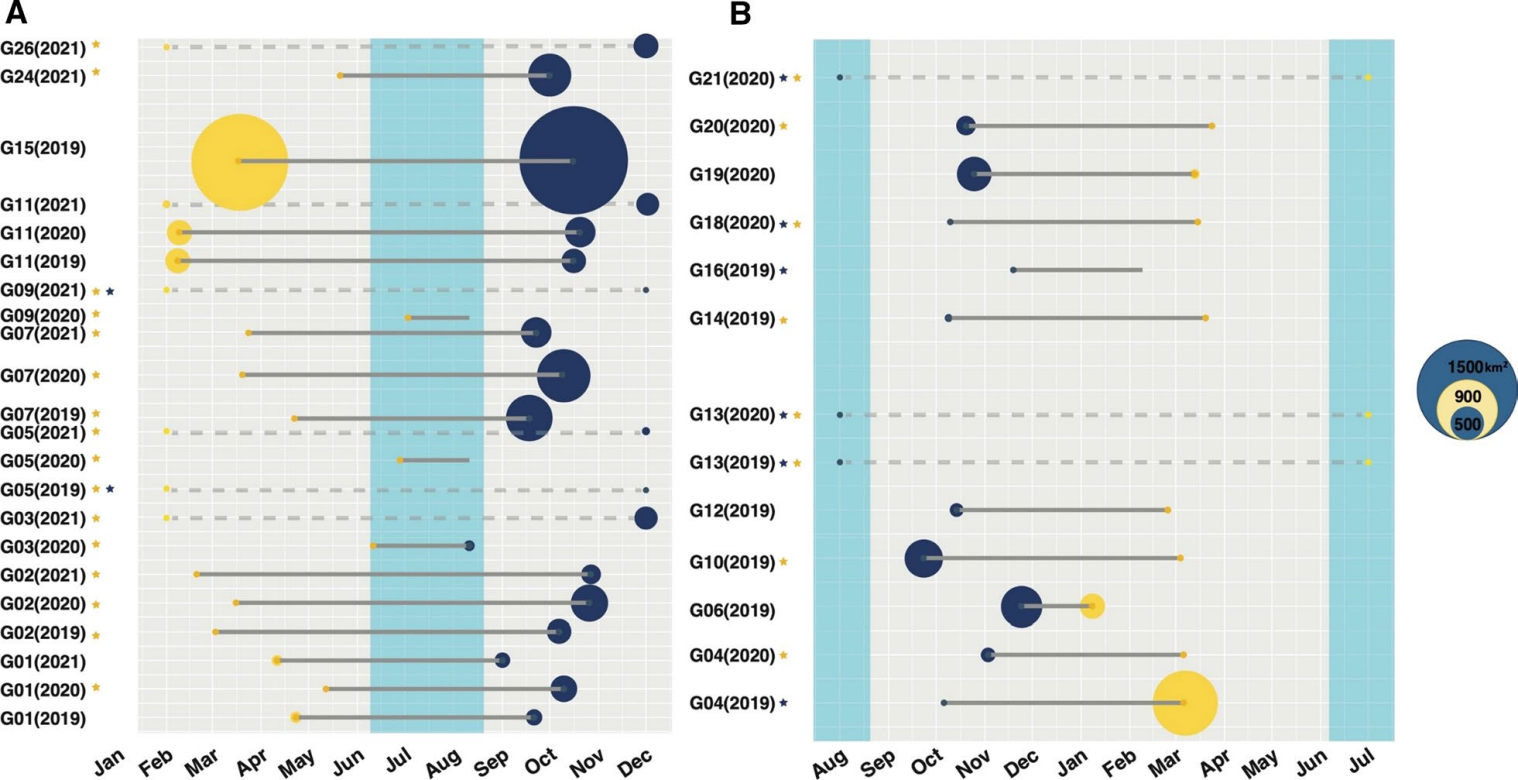
**A**–**B** Individual migration timelines for 21 adult guanacos (*Lama guanicoe*) collared in Patagonia, Argentina during 2019–2022. **A** Guanacos that started in their summer range and experienced winter migrations. **B** Individuals that started in their winter ranges and had a summer migration. Yellow dots correspond to winter migration departure date and blue dots indicate summer migration departure date. Grey bars indicate migration duration, if a dotted grey bar is present, the individual was classified as resident and is included to illustrate home range size. All timing parameters were derived from Net Squared Displacement analysis (NSD). Larger circle’s area are scaled to represent size of the 95% Autocorrelated Kernel Density Estimate (AKDE) home range of the corresponding season. Yellow circles are proportional to summer home ranges and blue circles to winter ones. Stars indicate individuals with home ranges that were too small to be noticeable if scaled (color of the star indicates season that is not scaled). If no circle is present, the individual did not have a delimited home range in that season. Light blue area indicates time of the year when snow cover is typically present in the study area. Circles provided for scale in km^2^

Spatial and temporal characteristics of migration derived from our NSD analysis exhibited high individual variability as well as seasonal trends (Additional file 1: Table S1). Mean migration timing from summer to winter ranges was March 21 in 2019 (range: Feb 8–Apr 22), April 3 in 2020 (range: Jan 7–Jul 27) and March 22 in 2021 (range: Feb 20–May 16). Mean migration timing from winter to summer ranges was October 10 in 2019 (range: Sep 12–Nov 18), October 1 in 2020 (range: Aug 9–Nov 2) and October 11 in 2021 (range: Sep 1–Nov 24). Individuals that were tracked for multiple migration cycles had similar migration timing dates (Fig. 2).

Migration distance ranged from altitudinal differences of 295–1106 m with all summer ranges occurring at higher altitudes than winter ones, which translated to distances that ranged from 5 to 50 km. We found the intra-individual variation for guanacos with multiple years of data was smaller than the inter-individual variation for all migratory guanacos for all migratory characteristics (Additional file 1: Table S2).

**Fig. 3:**
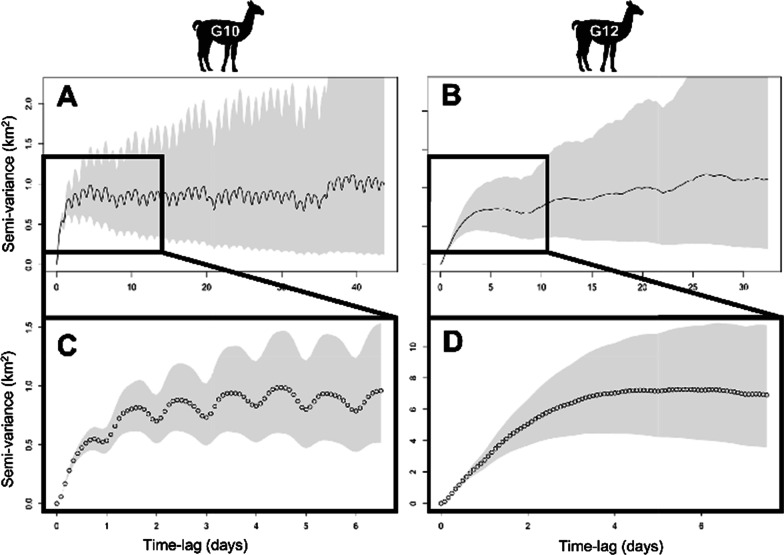
Individual variograms, illustrating the variability in distances between locations as a function of time for two individual (G10 and G12) adult guanacos (*Lama guanicoe*) in Patagonia, Argentina 2019–2022. Two temporal scales are shown: **A**–**B** illustrate one month of relocations; **C**–**D** the first 6 days of movement. Individual G10 (**A**, **C**) exhibited micro-migratory daily behaviors during its winter residence whereas individual G12 (**B**, **D**) did not

We observed considerable variation in the seasonal spatial use by guanacos (Additional file 1: Figs. S2 and S3). Some individuals had defined seasonal home ranges between which they alternated, while others had less constricted ranges. Indeed, for 9 of the migratory individuals this resulted in winter home ranges partially overlapping with their summer ones, leading to different behavioral classifications by our two methods (Additional file 1: Figs. S4 and S5). Despite this variation, home range size differed by season (χ2 = 27.31, *p* < 0.001) with winter home ranges ($\stackrel{-}{x }$= 354.6 km^2^; Median = 273.1; SD = 517.6) nearly 3× larger than summer ones ($\stackrel{-}{x }$= 120.0 km^2^; Median = 27.1; SD = 261.6) (Fig. 2). Home range fidelity was high both for winter ($\stackrel{-}{x }$ = 0.80; SD = 0.2) and summer ranges ($\stackrel{-}{x }$= 0.67; SD = 0.2). Guanacos moved at lower speeds within their winter ranges ($\stackrel{-}{x }$= 9.1 km/day; SD = 2) compared to summer ($\stackrel{-}{x }$= 10.3 km/day; SD = 3; χ2 = 5.2, *p* = 0.02), but they moved more during winter ($\stackrel{-}{x }$= 4.3 km/day; SD = 1.0) than in summer ($\stackrel{-}{x }$= 3.9 km/day; SD = 1.3; χ2 = 3.9, *p* = 0.04). However, we did not detect differences in overall distances moved between migratory and resident guanacos (t = − 0.78, df = 43, *p* = 0.4). We also found that at least 14 individuals had repetitive daily time-lag-dependent behaviors within some of their seasonal home ranges (Fig. 3). This occurred primarily in the summer (79%) for both migratory and resident individuals. Most guanacos that exhibited this behavior, did so for multiple seasons.

### Environmental drivers of migration–vegetation green-up and snow

**Fig. 4:**
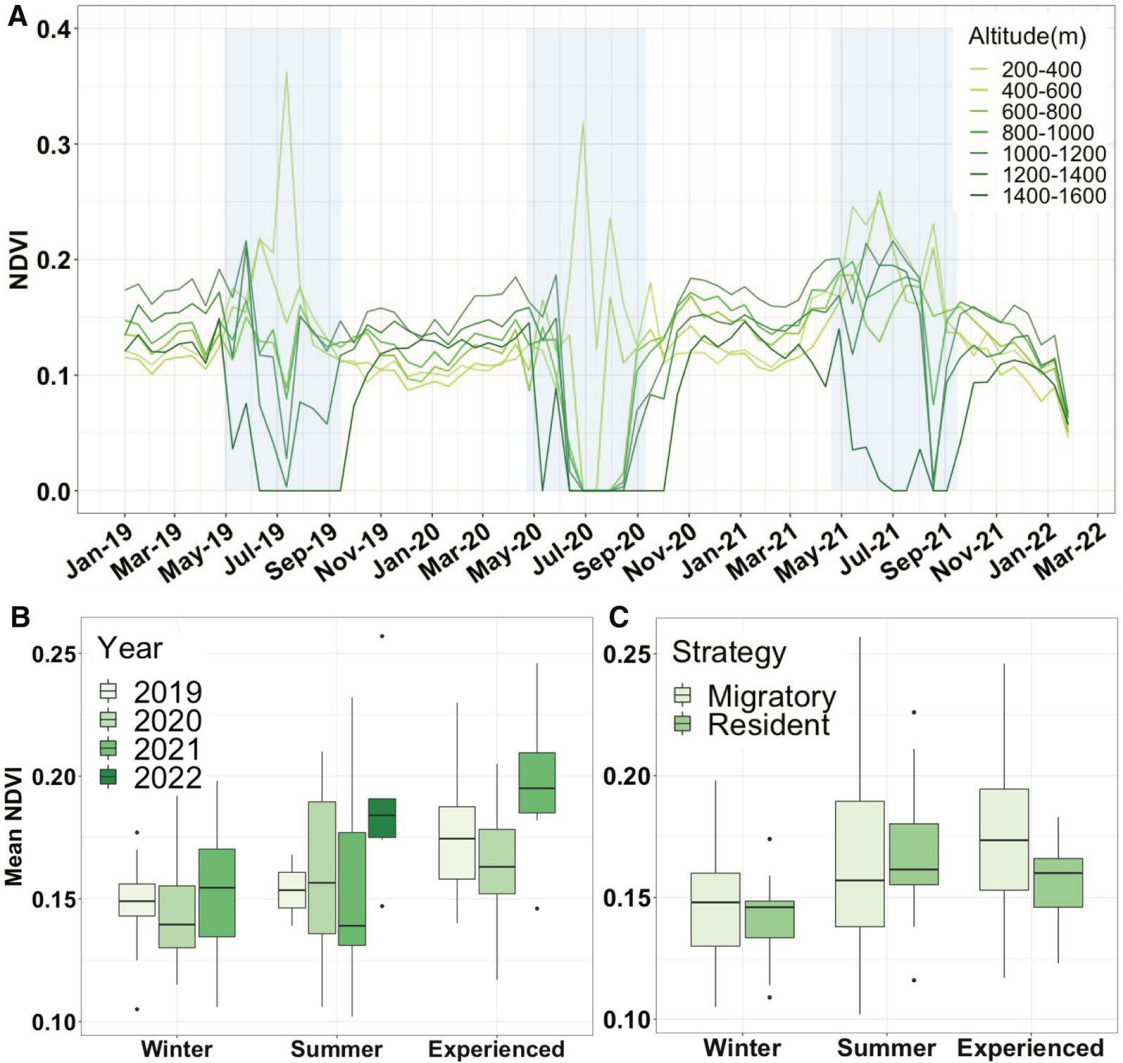
**A**) Mean normalized difference vegetation index (NDVI) curves starting winter 2019 to summer 2022, for 200 m altitudinal ranges covering the elevation gradient of the study area that includes the elevated Buenos Aires Plateau, in Patagonia, Argentina. Blue shaded areas correspond to the periods in which guanacos are present in their winter range located at lower altitudes. **B**–**C**) Boxplots showing mean cumulative NDVI values each individual would have experienced if it had remained in its winter or summer range, and the NDVI values it actually experienced throughout the year, **B** for each year of the study duration and **C** for migratory and non-migratory individuals

Phenology of the study area was highly correlated with altitude. Altitudes > 1000 m presented low and high NDVI values in winter and summer respectively, with summer’s up to 3.2× higher than in winter ($\stackrel{-}{x }$_summer_ = 0.13, SD = 0.02; $\stackrel{-}{x }$_winter_= 0.04, SD = 0.06 for the 1200–1600 m altitudinal range). However, elevations below 600 m presented an opposite pattern with NDVI peaking in winter, with winter mean up to 1.5× higher than summer mean ($\stackrel{-}{x }$_summer_ = 0.11, SD = 0.02; $\stackrel{-}{x }$_winter_ = 0.17, SD = 0.16 for the 200–600 m altitudinal range) (Fig. 4A). The lack of a uniform wave like progression of plant phenology through the study area prevented any sort of potential green wave surfing. However, guanacos were present in their summer ranges when NDVI peaked at higher altitudes, and migrated towards lower elevations during the winter, when the winter green-up occurred there (Fig. 4A). Additionally, migratory guanacos experienced higher levels of green-up compared to what they would have experienced if they had remained resident in either one of their seasonal ranges (χ2 = 26.2, df = 3, *p* < 0.001), with 18 migratory individuals’ paths (69%) having NDVI values that were higher than resident range values (Fig. 4B, C). Specifically, migratory guanacos cumulative NDVI was 8.4% higher than residents in their summer ranges, and 15.7% higher than residents in their winter ranges.

**Fig. 5:**
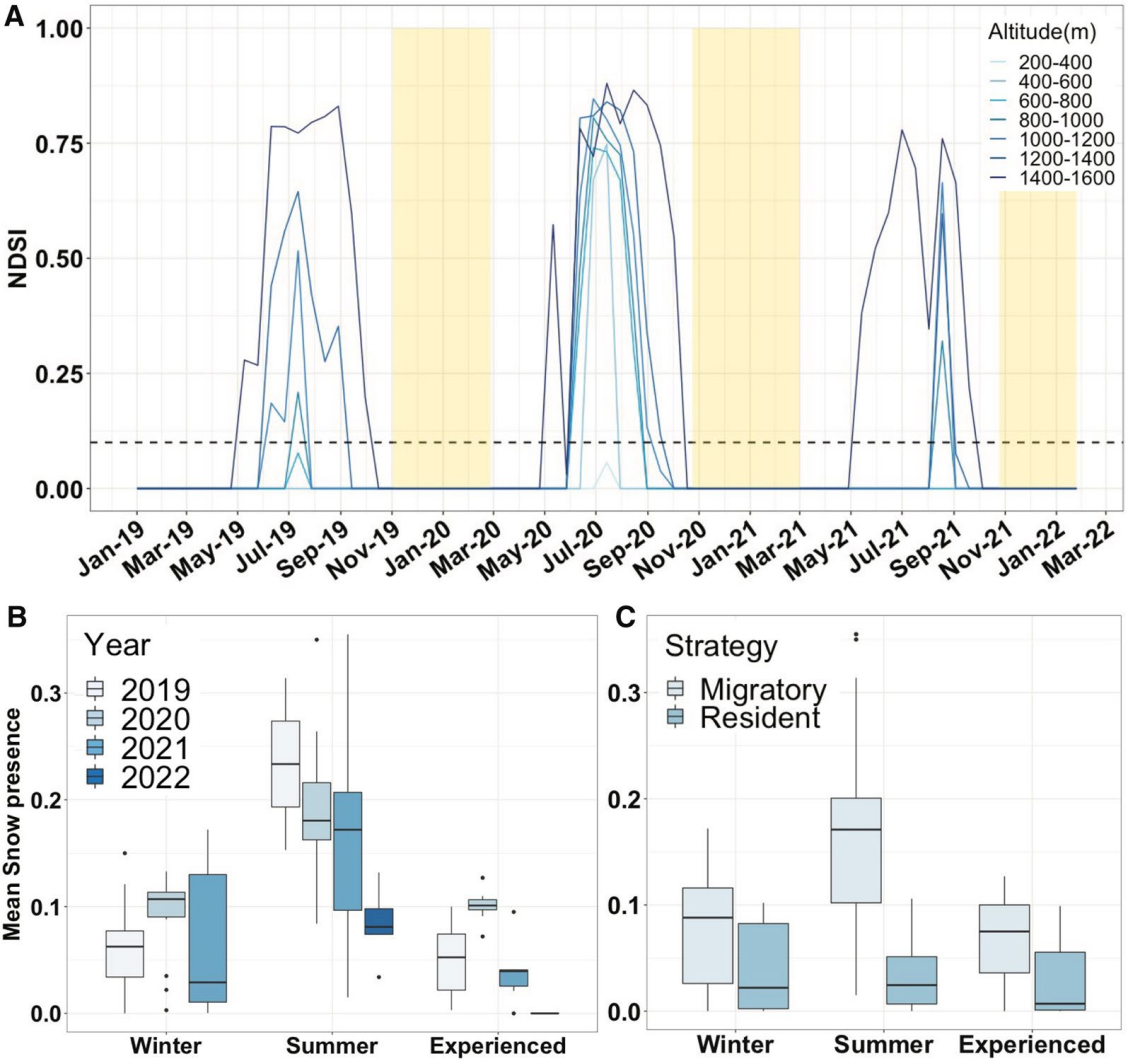
**A** Mean normalized difference snow index (NDSI) curves starting winter 2019 to summer 2022, for 200 m altitudinal ranges covering the elevation gradient of the study area that includes the elevated Buenos Aires Plateau, in Patagonia, Argentina. Yellow shaded areas indicate the periods in which guanacos are occupying their summer ranges at higher altitudes. The black dotted line (NDSI = 10) indicates the threshold above which we consider snow cover to be present in the area. **B**–**C** Boxplots showing mean cumulative snow cover presence values each individual would have experienced if it had remained in its winter or summer range, and the snow cover presence values it actually experienced throughout the year, **B** for each year of the study duration and **C** for migratory and non-migratory individuals

NDSI followed the same curve-shaped pattern throughout the altitudinal gradient with higher values during winter, but snow cover was present more consistently and for longer periods of time at higher elevations (Fig. 5A). Guanaco summer ranges coincided with the time of the year in which snow cover was absent in those higher altitudes, while spending winter at lower elevations, with shorter and more sporadic periods of snow cover (Fig. 5A). When comparing experienced versus theoretical snow cover presence values, we found migration paths also typically avoided areas with higher presence of snow (χ2 = 95.24 Df = 2, *p* < 0.001), since 17 migratory guanacos (65.4%) had lower NDSI values than they would have had if they had remained resident in either of their seasonal ranges (Fig. 5B, C). Migratory guanacos cumulative NDSI was 38.2% less than summer ranges and 14.5% less than winter ones.

## Discussion

The majority—nearly three-quarters—of the guanacos in the sampled population were migratory. These migrations were typified by relatively short distances along a steep elevational gradient. Similar to other ungulate species in mountainous landscapes [48], and previous observations of guanacos elsewhere [34, 49], guanacos in our study site spent the summer at elevations up 295–1105 m above their winter ranges. These elevational movements appeared to be influenced by both vegetation and snow phenology. Generally, migratory guanacos avoided snow-covered areas by occupying snow-free areas at lower altitudes during the winter. This strategy also provided access to higher-quality forage as guanaco’s use of seasonal ranges coincided with the peak of vegetation green-up in both winter and summer ranges. Although we were unable to directly evaluate temperature as a driver of migratory movements, it is worth noting that altitude appears to be a useful proxy for temperature given the correlation between the two (|r| = 0.9) in the study area. Furthermore, previous studies have found temperature to be less important than snow cover for ungulate migration, since individuals typically use areas with low temperatures as long as they are snow free [50].

Guanacos exhibited a combination of fidelity and plasticity regarding migratory characteristics. Even though our data on multiple migration periods was limited, we found individuals that displayed fidelity to migration (for up to three migratory cycles) as well as individuals that switched between migratory and resident strategies between consecutive years. Guanacos with multiple years of data that did not switch between strategies, tended to revisit the same summer and winter ranges, and exhibited similar residency times in their migratory ranges. With one exception, all guanacos that switched between strategies had summer ranges that, while still at higher altitudes than their winter ones, were not located in the high elevation plateau where most guanacos migrated to but were rather neighboring to their winter ranges. This change between strategies could be a result of guanacos often presenting exploratory movements outside of their home ranges, coupled with lower NDVI years that could force resident guanacos out of their usual boundaries for foraging purposes. We also observed important behavioral plasticity around the timing and duration of migration, both within and between individuals, with interindividual variation being highest. Indeed, migration departure dates differed by > 100 days, and duration of migration ranged from 1 to 21 days. Changes in migration timing could be related to annual variations in climatic conditions, as we found a trend for a later summer migration departure date in 2020, which registered a longer period of snow cover compared to 2019 and 2021, leading to a later snowmelt date.

Variation in migratory characteristics has also been described for a number of other ungulate species [51]. Typically, differences in migration timing, distance and duration have been associated with individual attributes, like age, sex, and reproductive status [16, 31, 52]. Although we did not detect notable differences between sexes, we did not account for guanacos´ age, nutritional and reproductive status. Given the tight social nature of guanacos that tend to migrate in family groups however, we would not expect major behavioral differences driven by individual attributes.

While most collared guanacos were migratory, we observed variation in migration propensity, with nine individual-years, corresponding to 7 guanacos, that remained resident in the winter ranges. This could be related to density-dependent resource release that follows departure of migratory individuals [48, 52], as well as to the absence of domestic cattle in the winter range that has been shown to overlap extensively with guanacos resulting in competition for resources [53, 54]. Partial migration appears to be common for guanacos across their distributional range [49, 53, 55]. Moreover, partial migration seems to be the predominant strategy among ungulates, with > 27 species exhibiting a mix of migratory and resident individuals within the same population [15, 52]. This strategy has been related to increased fitness and resilience, given the capacity of the population to respond to environmental changing conditions as well as to fluctuations in population density and predation risk [56]. Because pumas (*Puma concolor)*, guanacos´ main predator, have been found to not follow migratory individuals [49] and occur throughout the study area, and resident guanacos move as much as migratory ones within their home ranges, we do think neither predation risk nor energetic costs influence whether guanacos migrate. Instead, migratory guanacos likely benefit from accessing newly emerged vegetation, whereas resident guanacos benefit from reduced competition during the mating and breeding seasons. Resident guanacos may also reduce risks associated with encountering roads and fences during migration.

Multiple studies have shown that ungulate migrations follow certain patterns of plant phenology [22, 57]. For instance, the progression across the landscape of the instantaneous rate of green-up, a proxy of “springness” [26] can be compared to individual locations to evaluate whether animals are maximizing the use of newly emerged vegetation by surfing or jumping green waves [57–59]. However, we found that these wave-like patterns are not representative of the phenology in our study area. The Patagonian shrub and grass steppes exhibit a complex relationship between precipitation, temperature and annual variations in NDVI, with lower elevation areas presenting a lagged vegetative green-up that occurs in early winter [60, 61]. This corresponds with the pattern we detected in our study area, where higher elevations had wave like patterns with summer peaks while lower elevations had more unpredictable patterns with late fall and winter peaks. The particularities of the phenology along with the short migratory distances, therefore, likely hamper the ability of guanacos to surf green waves. However, we detected higher primary productivity levels in the greenscape experienced by migratory guanacos compared to the available greenscapes corresponding to their summer and winter ranges had they remained resident. Thus, although guanacos did not appear to surf green-waves, migratory guanacos did track general patterns of vegetation green-up.

Although less common than vegetation phenology, snow is also a major driver of ungulates migrations [59, 62–64]. Ungulates tend to avoid snow because increasing snow depth reduces mobility, exacts additional energetic costs, and hinders the search for forage, resulting in an overall poor nutritional condition [31]. As predicted, guanacos’ migration was associated to snow cover as they appeared to avoid “white waves”: migratory individuals occupied high altitude areas in spring and summer when snow was absent, but moved into low altitude areas featuring less now, during late fall and winter. Indeed, migratory individuals experienced less snow compared to what they would have experienced in their summer range. Having remained in their winter ranges in some cases however, would have meant even less exposure to snow, which could indicate that a synergy of both vegetation and snow phenology are driving guanacos’ migrations. In fact, given the strong correlation between these two variables, in which snow cover mediates vegetation green-up dynamics [65, 66], it is difficult to disentangle their effects at such spatial and temporal resolutions. This was the case in our study area, particularly so for high altitudes where NDSI reached higher values and snow cover was present for extended periods of time (Additional file 1: Fig. S8). Tracking vegetation waves has been identified as a primary cause behind ungulate migration [67, 68]. Our findings highlight the importance of disentangling other potential environmental factors driving migrations, particularly in arid environments where green waves may not occur. Given that ungulate migrations are declining globally [69], understanding the drivers behind these seasonal movements, as well as their spatial and temporal characteristics will be important for effective conservation of migratory ungulates.

Snow has also been related to smaller winter home ranges given the constraints it can have on animal movement [63, 70]. While we found that guanacos moved slower during the winter season, they had winter ranges that were larger than summer ones. Larger winter ranges are uncommon among migratory ungulates [50, 70–72] and could be related to snow depth being insufficient to constrain movement, or to a combination of factors including the fact that winter ranges are shared between migratory and resident individuals, as well as the fact that guanacos form larger social groups during this season [35] which could limit vegetation availability and force individuals to forage in larger areas. This is particularly relevant given that guanacos shift to browsing during winter which requires larger ranges due to the usual patchiness of this resource [73]. Snow cover could be a factor influencing winter home range size, but by reducing forage availability, as 2020 was both the year with higher snow presence cover and larger winter home ranges. Additionally, displaying male guanacos during the mating season might constrain movements and prevent access to certain areas leading to a reduction of the space effectively available in summer months [53]. Larger home ranges, particularly when covered by snow, may imply that animals are less selective of the areas they use, since they navigate the landscape following coarser-scale cues [72]. Consequently, animals appear to move more between different patches, leading to increased use of the landscape and even into the use of risky patches they would otherwise avoid.

While migration occurs at a landscape and seasonal level, driven by broad scale variables, such as snow and vegetation phenology, within seasonal ranges, habitat selection occurs at finer scales, driven by the daily need to maximize foraging while reducing predation risk and minimizing competition [2, 74]. We found that more than half of the collared guanacos exhibited daily micro-migrations nested within their seasonal ones. Such behaviors were more common during summer, and among residents (6/7 residents vs. 12/18 migrants) and resulted in small home ranges. Contrary to what we described for large winter home ranges, these micro-migrations seem to be in response to proximate cues and take advantage of landscape heterogeneity while prioritizing risk avoidance. These daily movements are possibly driven by local trade-offs between resource availability and perceived risk, as it has been found previously among vicuñas [75] that tended to visit highly productive but also high risk foraging sites during the day, while moving into lower productivity yet safer sites at night. Future work can explore the differences in nutritional state and predation events between guanacos that exhibit this daily behavior and the ones that do not to understand the benefits associated with these repetitive daily movements.

Guanacos exhibit a mixture of both fidelity and plasticity regarding migration, which might prove to be critical for the continuity of this strategy in the face of rapid environmental change. Climate change could negatively impact migration by creating a mismatch between climatic conditions, vegetation green-up and migratory movements. This in turn could decrease the benefits of migratory behaviors, which has been observed in other species of migratory ungulates [76]. Similarly, land use change and habitat fragmentation, such as those caused by roads and fences, may impact migratory movements by altering connectivity between seasonal ranges. Individual variations in temporal and spatial migration characteristics, including the ones we described for this guanaco population, could provide the necessary plasticity for this species to adapt to changing environmental conditions.

## Conclusion

Understanding of where, when, and why animals migrate is important to assess potential adaptations to future scenarios and to inform conservation policies to protect summer and winter ranges as well as corridors that connect them. We found guanacos have altitudinal migrations that track vegetation and snow phenology, displaying a combination of fidelity and flexibility in the temporal and spatial characteristics of their movements. Provided that habitat and connectivity are maintained, and climate change does not outpace the capacity of this species to adapt, the high degree of plasticity in guanaco migration has the potential to buffer these effects. Altering migration timing or location of their seasonal ranges can ensure guanacos continue to match vegetation green-up and snow patterns to their seasonal habitat use, allowing this species the capacity to respond to change without losing their ancestral seasonal migrations.

## Supplementary Information

**Additional file 1: Fig. S1.** Net square displacement plots of each migratory cycle for 3 guanacos (G01, G02, G04) that remained migratory for the duration of the study showing all three a priori models (migrant, disperser and resident) fit to the individual’s relocations (black points were used fit the migratory model, grey points were not due to poor fit), with their corresponding AIC value, and the distribution and classification of each relocation to either the starting range (range 1, red points), their migratory range (range 2, blue points) or unclassified locations (grey points).** Fig. S2.** Net square displacement plots for 2 individual guanacos (G11, G13) that were originally classified as migratory showing all three a priori models (migrant, disperser and resident) fit to the individual’s relocations (black points were used fit the migratory model, grey points were not due to poor fit), with their corresponding AIC value, and the distribution and classification of each relocation to either the starting range (range 1, red points), their migratory range (range 2, blue points) or unclassified locations (grey points). When incorporating the home range overlap method, G11 was considered to have switched strategies in 2021 and was finally classified as resident, and G13 was considered resident for both cycles.** Fig. S3.** Standard (distance) and elevation Net Square Displacement (NSD) plots for 2 individual guanacos (G01, G11) to visually represent differences in model fit and grouping of locations into two seasonal ranges, showing all a priori models (migrant, mixed-migrant, disperser, nomad and resident) with their corresponding AIC value. Black points correspond to the locations that are used to fit the migratory model, grey points are locations that are discarded due to their poor fit.** Fig. S4.** GPS locations and 95% Kernel Density Estimate (KDE) home ranges with color gradients for three guanacos classified as migratory by the home range overlap method (G01, G02, G04) illustrating summer (lighter) and winter (darker) ranges with different degrees of overlap and Bhattacharyya's affinity index (BA = 0 for all migratory cycles of G02 and G04; 0 > BA ≥ 0.15 for all migratory cycles of G01).** Fig. S5.** GPS locations and 95% Kernel Density Estimate (KDE) home ranges with color gradients for two guanacos (G11, G13) illustrating summer (lighter) and winter (darker) ranges with different degrees of overlap and Bhattacharyya's affinity index. G11 was classified as migratory by the home range overlap method for 2019 (0 > BA ≥ 0.15) but was classified as resident for 2020 and 2021 (BA ≤ 0.15). G13 was classified as resident for both cycles (BA ≤ 0.15).** Fig. S6.** Summer mean NDVI values for all pixels that fall under different landcover types in our study area. Landcover types were obtained from the European Space Agency (ESA) based on Sentinel-1 and Sentinel-2 data at 10m resolution. Wetland had the highest NDVI values ($(\bar{x})$ = 45.63, SD = 11.2) followed by shrublands ($(\bar{x})$ = 18.58, SD = 8.9) and grasslands ($(\bar{x})$ = 14.25, SD = 6.6) and was lowest in barren/scarce vegetation ($(\bar{x})$ = 11.148, SD = 4.6).** Fig. S7.** Schematic representation of the space-time-time matrix, where rows represent each location the individual used (li); columns represent days (ti) that span throughout the duration of our study; and the diagonal (l(tT)(tT)) represents the day the animal was actually present at the corresponding location.** Fig. S8.** Plots of raw Normalized Difference Snow Index (NDSI) and Normalized Difference Vegetation Index (NDVI) summer 2019 to summer 2022, for 200 m altitudinal ranges covering the elevation gradient of the study area, in Patagonia, Argentina.** Table S1.** Attributes of migration for 21 adult guanacos (Lama guanicoe) equipped with GPS collars in Patagonia, Argentina 2019-2022. Identification of seasonal behaviors (migratory, residential or dispersal) according to two methods, altitudinal Net Squared Displacement (NSD) and Seasonal Range Overlap, final classification and migration parameters regarding timing, duration and distance for individuals classified as migratory obtained from the top NSD model.** Table S2.** Intraindividual coefficient of variation for migratory guanacos with multiple years of data (3 individuals with 3 years of data and 2 individuals with 2 years of data) compared to intraindividual variation for all collared guanacos for different migratory characteristics.

**Additional file 2**. Net squared displacement raw data corresponding to 5 guanaco migratory periods, 3 summers (2019-2021) and 2 winters (2019-2020).

## Data Availability

The datasets supporting the conclusions of this article are included within the article and its additional files.

## Abbreviations

AKDE: Autocorrelated Kernel density estimate
AICc: Akaike’s information criterion
BA: Bhattacharyya’s affinity index
KDE: Kernel density estimate
NDSI: Normalized difference snow index
NDVI: Normalized difference vegetation index
NSD: Net squared displacement

## Code availability

All code used is available from the corresponding author upon request.

## References

[1] Boyce MS (1979). Seasonality and patterns of natural selection for life histories. Am Nat.

[2] Anderson KJ, Jetz W (2005). The broad-scale ecology of energy expenditure of endotherms. Ecol Lett.

[3] Pauli JN, Zuckerberg B, Whiteman JP, Porter W (2013). The subnivium: a deteriorating seasonal refugium. Front Ecol Environ.

[4] Dingle H, Drake A (2007). What Is migration?. BioScience.

[5] Berger J (2004). The last mile: how to sustain long-distance migration in mammals. Conserv Biol.

[6] Sinclair ARE. The function of distance movements in vertebrates. In: Swingland R, Greenwood PJ, editors. Ecol Anim Mov. Oxford, United Kingdom: Oxford University Press; 1983. p. 248–58.

[7] Avgar T, Street G, Fryxell JM (2014). On the adaptive benefits of mammal migration. Can J Zool.

[8] Middleton AD, Cook JG, Nelson AA, McWhirter DE, Klaver RW, Kauffman MJ (2013). Animal migration amid shifting patterns of phenology and predation: lessons from a Yellowstone elk herd. Ecol Soc Am.

[9] Fryxell JM, Sinclair ARE (1988). Causes and consequences of migration by large herbivores. Trends Ecol Evol.

[10] Rickbeil GJM, Merkle JA, Anderson G, Atwood MP, Beckmann JP, Cole EK (2019). Plasticity in elk migration timing is a response to changing environmental conditions. Glob Change Biol.

[11] Sawyer HM, Kauffman MJ, Nielson RM, Horne JS (2009). Identifying and prioritizing ungulate migration routes for landscape-level conservation. Ecol Appl.

[12] Sawyer H, Merkle JA, Middleton AD, Dwinnell SPH, Monteith KL (2019). Migratory plasticity is not ubiquitous among large herbivores. J Anim Ecol.

[13] Peters W, Hebblewhite M, Mysterud A, Eacker D, Hewison AJM, Linnell JDC (2019). Large herbivore migration plasticity along environmental gradients in Europe: life-history traits modulate forage effects. Oikos.

[14] Chapman BB, Brönmark C, Jan-Åke N, Lars-Anders H (2011). The ecology and evolution of partial migration. Oikos.

[15] Xu W, Barker K, Shawler A, Van Scoyoc A, Smith JA, Mueller T (2021). The plasticity of ungulate migration in a changing world. Ecology.

[16] Eggeman SL, Hebblewhite M, Bohm H, Whittington J, Merrill EH (2016). Behavioural flexibility in migratory behaviour in a long-lived large herbivore. J Anim Ecol.

[17] Kaitala A, Kaitala V, Lundberg P (1993). A theory of partial migration. Am Nat.

[18] Fuller TK, Keith LB (1981). Woodland caribou population dynamics in northeastern Alberta. J Wildl Manag.

[19] Hebblewhite M, Merrill EH (2011). Demographic balancing of migrant and resident elk in a partially migratory population through forage-predation tradeoffs. Oikos.

[20] Singh NJ, Grachev IA, Bekenov AB, Milner-Gulland EJ (2010). Tracking greenery across a latitudinal gradient in central Asia–the migration of the saiga antelope. Divers Distrib.

[21] Teitelbaum CS, Fagan WF, Fleming CH, Dressler G, Calabrese JM, Leimgruber P (2015). How far to go? determinants of migration distance in land mammals. Ecol Lett.

[22] Merkle JA, Monteith KL, Aikens EO, Hayes MM, Hersey KR, Middleton AD (2016). Large herbivores surf waves of green-up during spring. Proc R S.

[23] Van der Graaf AJ, Stahl J, Klimkowska A, Bakker JP, Drent RH (2006). Surfing on a green wave-how plant growth drives spring migration in the Barnacle Goose Branta leucopsis. Ardea.

[24] Fryxell JM, Greever J, Sinclair ARE (1988). Why are migratory ungulates so abundant?. Am Nat.

[25] Hebblewhite M, Merrill EH, McDermid G (2008). A multi-scale test of the forage maturation hypothesis in a partially migratory ungulate population. Ecol Monogr.

[26] Bischof R, Loe LE, Meisingset EL, Zimmermann B, van Moorter B, Mysterud A (2012). A migratory Northern ungulate in the pursuit of spring: jumping or surfing the green wave?. Am Nat.

[27] Monteith KL, Bleich VC, Stephenson TR, Pierce BM, Conner MM, Klaver RW (2011). Timing of seasonal migration in mule deer: effects of climate, plant phenology, and life-history characteristics. Ecosphere.

[28] Brinkman TJ, Deperno CS, Jenks JA, Haroldson BS, Osborn RG (2005). Movement of female white-tailed deer: effects of climate and intensive row-crop agriculture. J Wildl Manag.

[29] Rivrud IM, Bischof R, Meisingset EL, Zimmermann B, Loe LE, Mysterud A (2016). Leave before it’s too late: anthropogenic and environmental triggers of autumn migration in a hunted ungulate population. Ecology.

[30] Peng S, Piao S, Ciais P, Fang J, Wang X (2010). Change in winter snow depth and its impacts on vegetation in China. Glob Chang Biol.

[31] Singh NJ, Börger L, Dettki H, Bunnefeld N, Ericsson G (2012). From migration to nomadism: movement variability in a northern ungulate across its latitudinal range. Ecol Appl.

[32] Franklin WL (1982). Biology, ecology, and relationship to man of the South American camelids. Mamm Biol S Am.

[33] Wurstten A, Novaro AJ, Walker RS (2014). Habitat use and preference by guanacos, vicuñas, and livestock in an altitudinal gradient in northwest Argentina. Eur J Wildl Res.

[34] Mueller T, Olson KA, Dressler G, Leimgrube P, Fuller TK, Nicolson C (2011). How landscape dynamics link individual to population-level movement patterns: a multispecies comparison of ungulate relocation data. Glob Ecol Biogeogr.

[35] Ortega IM, Franklin WL (1995). Social organization, distribution and movements of a migratory guanaco population in the Chilean Patagonia. Rev Chil de Hist Nat.

[36] Bertiller MB, Beeskow AM, Coronato F (1991). Seasonal environmental variation and plant phenology in arid Patagonia (Argentina). J Arid Environ.

[37] Cabrera AL (1971). Fitogeografía de la República Argentina. Bol de la Soc Argent de Bot.

[38] Movia CP, Soriano A, Leon RJC. La vegetación de la cuenca del río Santa Cruz (provincia de Santa Cruz, Argentina). Instit de Bot Darwinion (Darwininana; vols. 1–4); 1987; 9–78.

[39] R Core Team. A language and environment for statistical computing [Internet]. Vienna, Austria; 2016. Available from: https://www.R-project.org/.

[40] Bunnefeld N, Börger L, van Moorter B, Rolandsen C, Dettki H, Solberg EJ (2011). A model-driven approach to quantify migration patterns: individual, regional and yearly differences. J Anim Ecol.

[41] Spitz DB, Hebblewhite M, Stephenson TR (2017). MigrateR: extending model-driven methods for classifying and quantifying animal movement behavior. Ecography.

[42] Cagnacci F, Focardi S, Ghisla A, van Moorter B, Merrill EH, Gurarie E (2016). How many routes lead to migration? comparison of methods to assess and characterize migratory movements. J Anim Ecol.

[43] Calenge C (2006). The package “adehabitat” for the R software: a tool for the analysis of space and habitat use by animals. Ecol Model.

[44] Worton BJ (1989). Kernel methods for estimating the utilization distribution in home-range studies. Ecology.

[45] Calabrese JM, Fleming CH, Gurarie E (2016). ctmm: an R package for analyzing animal relocation data as continuous-time stochastic process. Methods Ecol Evol.

[46] Bates D, Mächler M, Bolker B, Walker S (2015). Fitting linear mixed-effects models using lme4. J Stat Softw.

[47] Gudex-Cross D, Keyser SR, Zuckerberg B, Fink D, Zhu L, Pauli JN (2021). Winter habitat indices (WHIs) for the contiguous US and their relationship with winter bird diversity. Remote Sens Environ.

[48] Mysterud A (1999). Seasonal migration pattern and home range of roe deer (Capreolus capreolus) in an altitudinal gradient in southern Norway. J Zool.

[49] Gelin ML, Branch LC, Thornton DH, Novaro AJ, Gould MJ, Caragiulo A (2017). Response of pumas (Puma concolor) to migration of their primary prey in Patagonia. PLoS ONE.

[50] Zweifel-Schielly B, Kreuzer M, Ewald KC, Suter W (2009). Habitat selection by an Alpine ungulate: the significance of forage characteristics varies with scale and season. Ecography.

[51] Guan TP, Ge BM, McShea WJ, Li S, Song YL, Stewart CM (2013). Seasonal migration by a large forest ungulate: a study on takin (Budorcas taxicolor) in Sichuan province, China. Eur J Wildl Res.

[52] Berg JE, Hebblewhite M, St. Clair CC, Merrill EH (2019). Prevalence and mechanisms of partial migration in ungulates. Front Ecol Evol.

[53] Moraga CA, Funes MC, Pizarro JC, Briceño C, Novaro AJ (2015). Effects of livestock on guanaco Lama guanicoe density, movements and habitat selection in a forest–grassland mosaic in Tierra del Fuego, Chile. Oryx.

[54] Puig S, Videla F, Cona MI, Monge SA (2001). Use of food availability by guanacos (Lama guanicoe) and livestock in Northern Patagonia (Mendoza, Argentina). J Arid Environ.

[55] Schroeder NM, Matteucci SD, Moreno PG, Gregorio P, Ovejero R, Taraborelli P (2014). Spatial and seasonal dynamic of abundance and distribution of guanaco and livestock: insights from using density surface and null models. PLoS ONE.

[56] Mysterud A, Loe LE, Zimmermann B, Bischof R, Veiberg V, Meisingset E (2011). Partial migration in expanding red deer populations at northern latitudes–a role for density dependence?. Oikos.

[57] Aikens EO, Mysterud A, Merkle JA, Cagnacci F, Rivrud IM, Hebblewhite M (2020). Wave-like patterns of plant phenology determine ungulate movement tactics. Curr Biol.

[58] Sawyer H, Kauffman MJ (2011). Stopover ecology of a migratory ungulate. J Anim Ecol.

[59] Laforge MP, Bonar M, Vander Wal E (2021). Tracking snowmelt to jump the green wave: phenological drivers of migration in a northern ungulate. Ecology.

[60] Bianchi E, Villalba R, Solarte A (2020). NDVI spatio-temporal patterns and climatic controls over northern Patagonia. Ecosystems.

[61] Bruzzone O, Easdale MH (2021). Archetypal temporal dynamics of arid and semi-arid rangelands. Remote Sens Environ.

[62] Ball JP, Nordengren C, Wallin K (2001). Partial migration by large ungulates: characteristics of seasonal moose Alces alces ranges in northern Sweden. Wildl Biol.

[63] Luccarini S, Mauri L, Ciuti S, Lamberti P, Apollonio M (2006). Red deer (Cervus elaphus) spatial use in the Italian Alps: home range patterns, seasonal migrations, and effects of snow and winter feeding. Ethol Ecol Evol.

[64] Severson JP, Johnson HE, Arthur SM, Leacock WB, Suitor MJ (2021). Spring phenology drives range shifts in a migratory arctic ungulate with key implications for the future. Glob Change Biol.

[65] Wang X, Wu C, Peng D, Gonsamo A, Liu Z (2018). Snow cover phenology affects alpine vegetation growth dynamics on the Tibetan plateau: satellite observed evidence, impacts of different biomes, and climate drivers. Agric For Meteorol.

[66] Xiong T, Zhang H, Zhao J, Zhang Z, Guo X, Zhu Z (2019). Diverse responses of vegetation dynamics to snow cover phenology over the boreal region. Forests.

[67] Mysterud A, Bischof R, Loe LE, Odden J, Linnell JDC (2012). Contrasting migration tendencies of sympatric red deer and roe deer suggest multiple causes of migration in ungulates. Ecosphere.

[68] Abraham JO, Upham NS, Damian-Serrano A, Jesmer BR (2022). Evolutionary causes and consequences of ungulate migration. Nat Ecol Evol.

[69] Harris G, Thirgood S, Hopcraft JGC, Cromsigt JPGM, Berger J (2009). Global decline in aggregated migrations of large terrestrial mammals. Endanger Species Res.

[70] Shakeri YN, White KS, Waite JN (2021). Staying close to home: ecological constraints on space use and range fidelity in a mountain ungulate. Ecol Evol.

[71] Grignolio S, Rossi I, Bassano B, Apollonio M (2007). Predation risk as a factor affecting sexual segregation in Alpine Ibex. J Mamm.

[72] Pearson SM, Turner MG, Wallace LL, Romme WH (1995). Winter habitat use by large ungulates following fire in Northern Yellowstone national park. Ecol Appl.

[73] Ofstad EG, Herfindal I, Solberg EJ, Sæther BE (2016). Home ranges, habitat and body mass: simple correlates of home range size in ungulates. Proc R Soc B Biol Sci.

[74] Bose S, Forrester TD, Casady DS, Wittmer HU (2018). Effect of activity states on habitat selection by black-tailed deer. J Wildl Manag.

[75] Smith JA, Donadio E, Pauli JN, Sheriff MJ, Middleton AD (2019). Integrating temporal refugia into landscapes of fear: prey exploit predator downtimes to forage in risky places. Oecologia.

[76] Aikens EO, Monteith KL, Merkle JA, Fralick GL, Kauffman MJ (2020). Drought reshuffles plant phenology and reduces the foraging benefit of green-wave surfing for a migratory ungulate. Glob Change Biol.

