# Supplementary material for: Phenological drivers of ungulate migration in South America: characterizing the movement and seasonal habitat use of guanacos
Source: Mov Ecol. 2022 Aug 13;10:34. doi: 10.1186/s40462-022-00332-7 (PMC9375948; doi:10.1186/s40462-022-00332-7)
Supplement: Supplementary file 1 — Additional file 1: Fig. S1. Net square displacement plots of each migratory cycle for 3 guanacos (G01, G02, G04) that remained migratory for the duration of the study showing all three a priori models (migrant, disperser and resident) fit to the individual’s relocations (black points were used fit the migratory model, grey points were not due to poor fit), with their corresponding AIC value, and the distribution and classification of each relocation to either the starting range (range 1, red points), their migratory range (range 2, blue points) or unclassified locations (grey points). Fig. S2. Net square displacement plots for 2 individual guanacos (G11, G13) that were originally classified as migratory showing all three a priori models (migrant, disperser and resident) fit to the individual’s relocations (black points were used fit the migratory model, grey points were not due to poor fit), with their corresponding AIC value, and the distribution and classification of each relocation to either the starting range (range 1, red points), their migratory range (range 2, blue points) or unclassified locations (grey points). When incorporating the home range overlap method, G11 was considered to have switched strategies in 2021 and was finally classified as resident, and G13 was considered resident for both cycles. Fig. S3. Standard (distance) and elevation Net Square Displacement (NSD) plots for 2 individual guanacos (G01, G11) to visually represent differences in model fit and grouping of locations into two seasonal ranges, showing all a priori models (migrant, mixed-migrant, disperser, nomad and resident) with their corresponding AIC value. Black points correspond to the locations that are used to fit the migratory model, grey points are locations that are discarded due to their poor fit. Fig. S4. GPS locations and 95% Kernel Density Estimate (KDE) home ranges with color gradients for three guanacos classified as migratory by the home range overlap method (G01, G02, G [file 40462_2022_332_MOESM1_ESM.docx]

**Phenological drivers of ungulate migration in South America: Characterizing the movement and seasonal habitat use of guanacos.**

Malena Candino^1^, Emiliano Donadio^2^, Jonathan N. Pauli^1^

^1^Department of Forest and Wildlife Ecology, University of Wisconsin, Madison, WI 53706, U.S.A.

^2^Fundación Rewilding Argentina, Buenos Aires, Argentina. CP 1425.

**Corresponding author:** Malena Candino, [candino@wisc.edu](mailto:candino@wisc.edu), 608-209-0291.


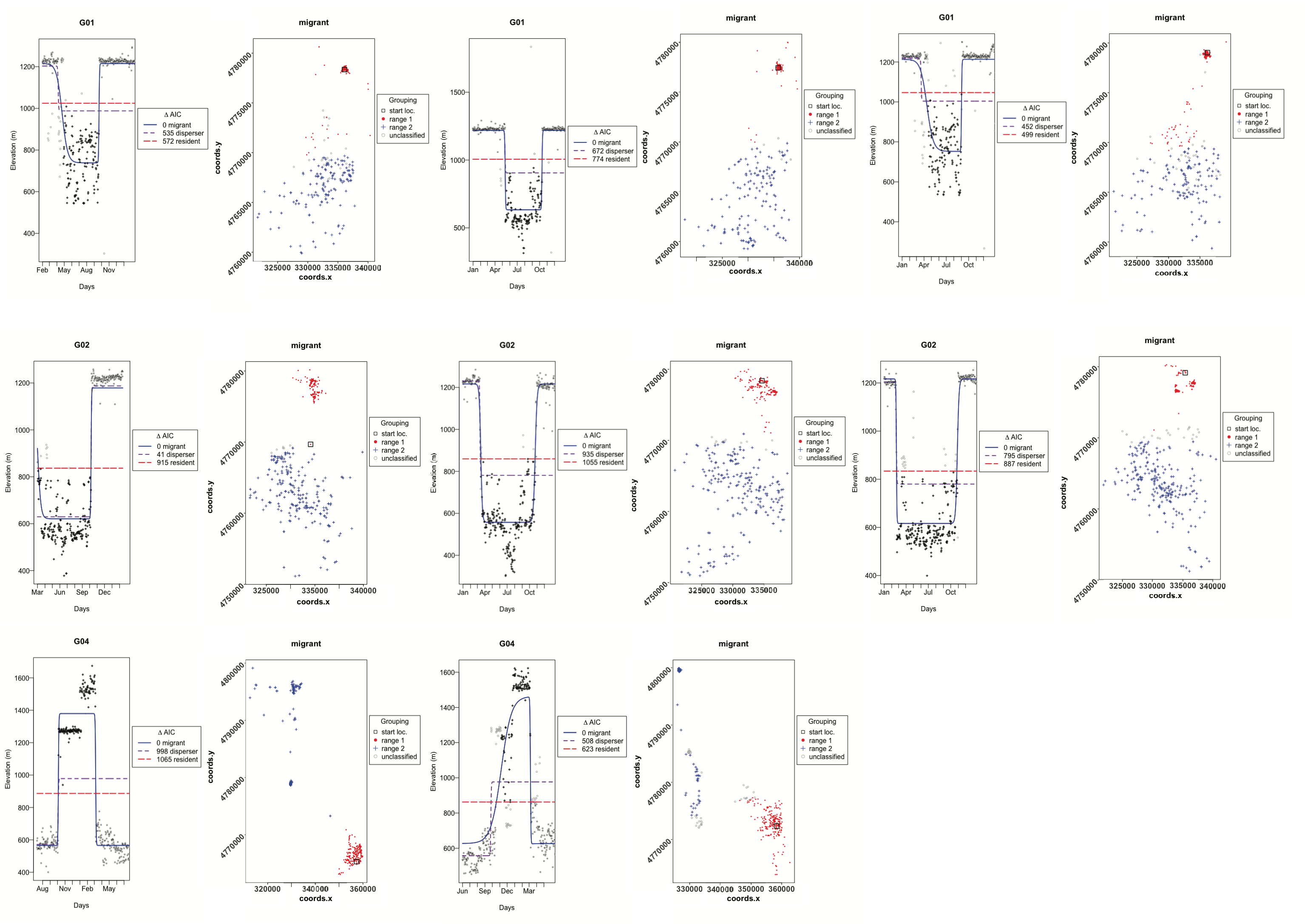


Figure 1. Net square displacement plots of each migratory cycle for 3 guanacos (G01, G02, G04) that remained migratory for the duration of the study showing all three a priori models (migrant, disperser and resident) fit to the individual’s relocations (black points were used fit the migratory model, grey points were not due to poor fit), with their corresponding AIC value, and the distribution and classification of each relocation to either the starting range (range 1, red points), their migratory range (range 2, blue points) or unclassified locations (grey points).


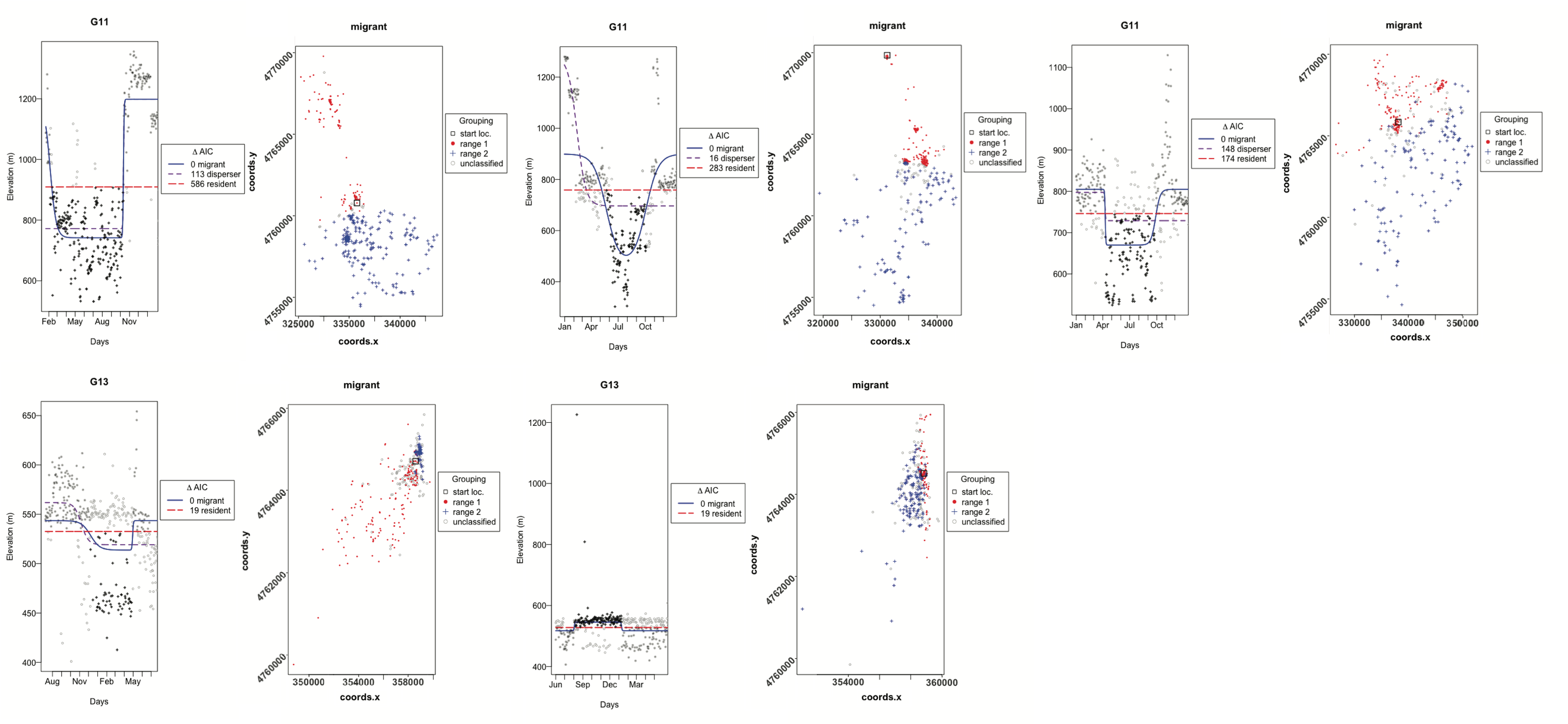


Figure 2. Net square displacement plots for 2 individual guanacos (G11, G13) that were originally classified as migratory showing all three a priori models (migrant, disperser and resident) fit to the individual’s relocations (black points were used fit the migratory model, grey points were not due to poor fit), with their corresponding AIC value, and the distribution and classification of each relocation to either the starting range (range 1, red points), their migratory range (range 2, blue points) or unclassified locations (grey points). When incorporating the home range overlap method, G11 was considered to have switched strategies in 2021 and was finally classified as resident, and G13 was considered resident for both cycles.


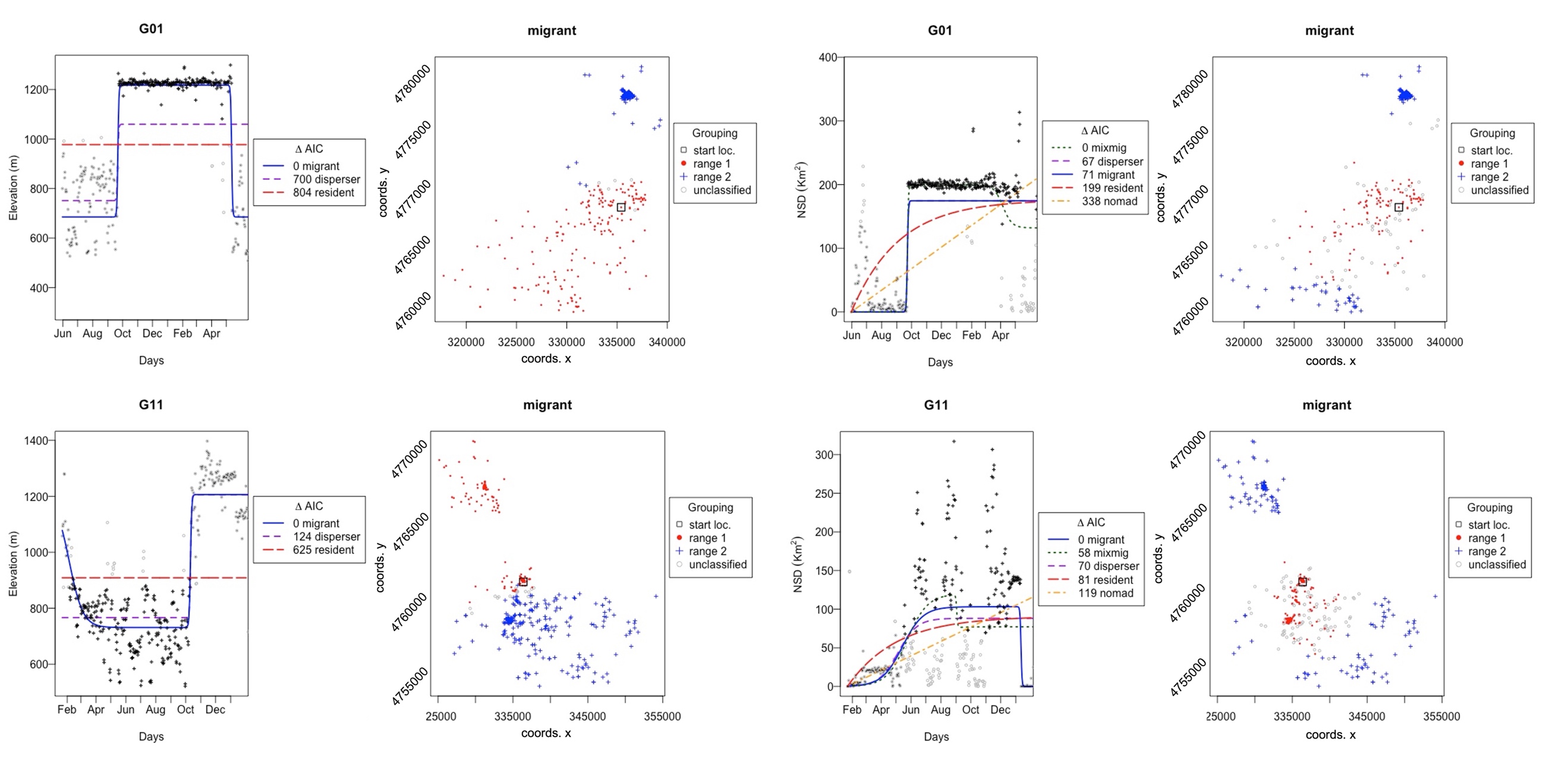


Figure 3. Standard (distance) and elevation Net Square Displacement (NSD) plots for 2 individual guanacos (G01, G11) to visually represent differences in model fit and grouping of locations into two seasonal ranges, showing all a priori models (migrant, mixed-migrant, disperser, nomad and resident) with their corresponding AIC value. Black points correspond to the locations that are used to fit the migratory model, grey points are locations that are discarded due to their poor fit.


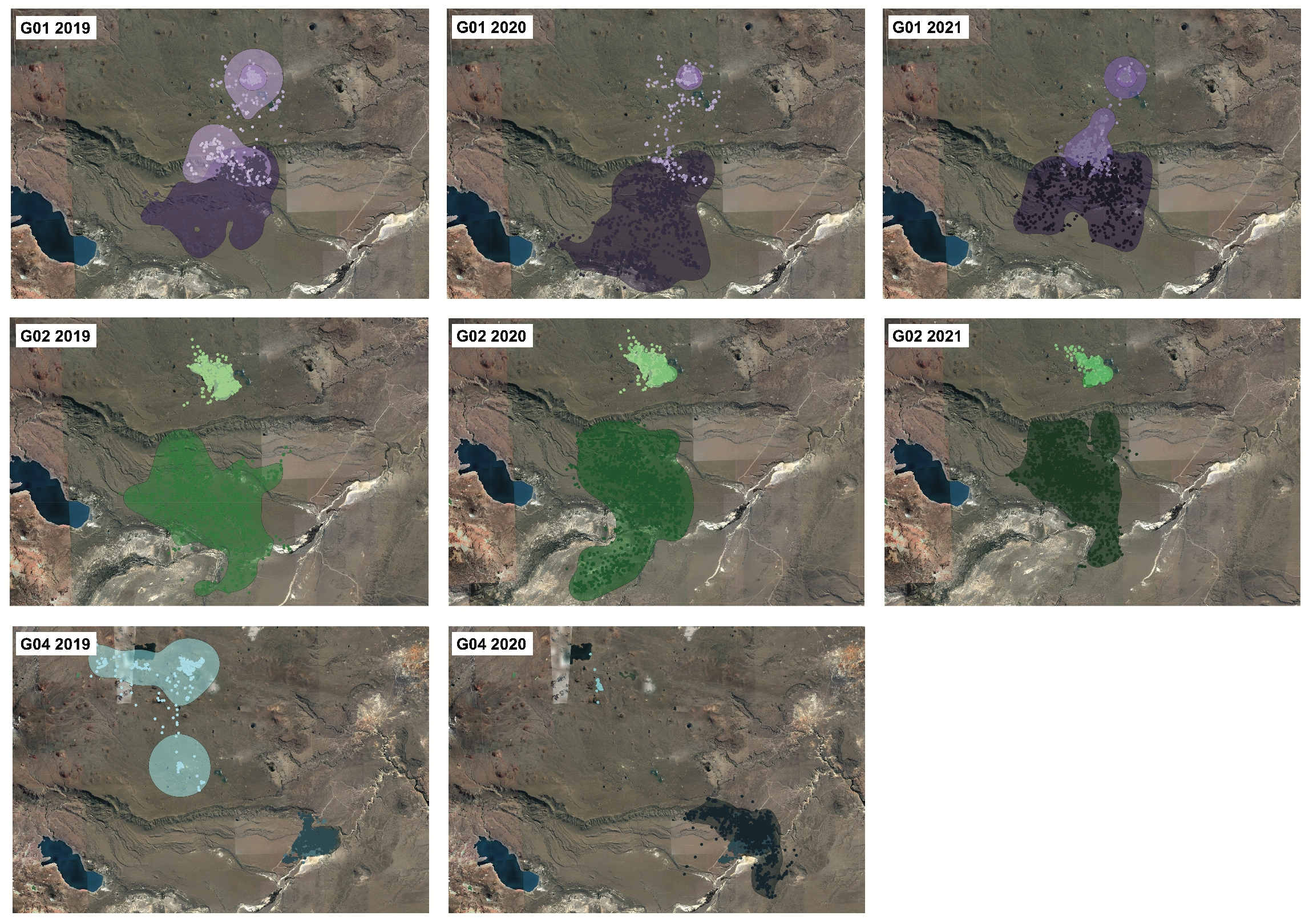


Figure 4. GPS locations and 95% Kernel Density Estimate (KDE) home ranges with color gradients for three guanacos classified as migratory by the home range overlap method (G01, G02, G04) illustrating summer (lighter) and winter (darker) ranges with different degrees of overlap and Bhattacharyya's affinity index (BA = 0 for all migratory cycles of G02 and G04; 0>BA≥0.15 for all migratory cycles of G01).


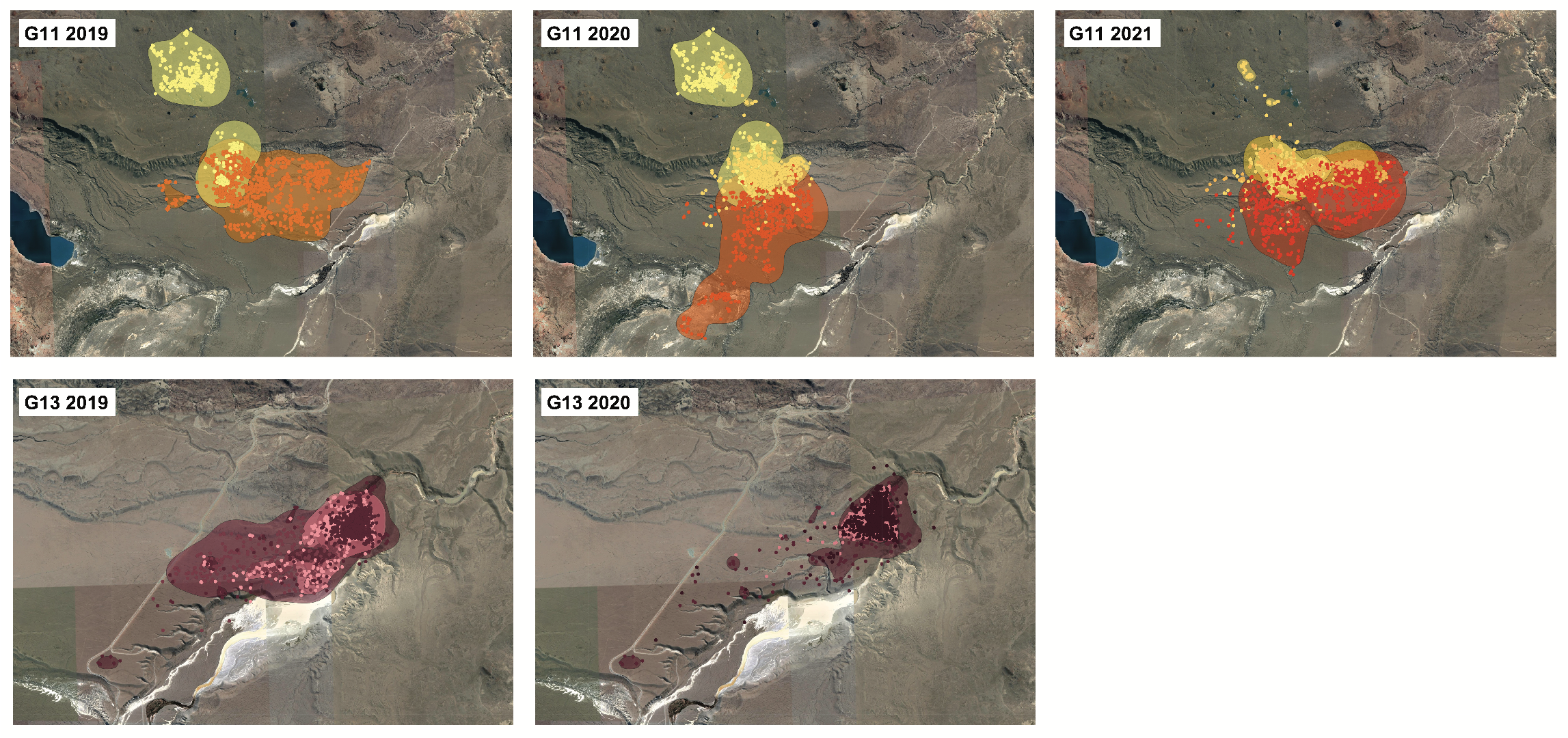


*Figure 5*. GPS locations and 95% Kernel Density Estimate (KDE) home ranges with color gradients for two guanacos (G11, G13) illustrating summer (lighter) and winter (darker) ranges with different degrees of overlap and Bhattacharyya's affinity index. G11 was classified as migratory by the home range overlap method for 2019 (0>BA≥0.15) but was classified as resident for 2020 and 2021 (BA ≤0.15). G13 was classified as resident for both cycles (BA ≤0.15).

Figure 6. Summer mean NDVI values for all pixels that fall under different landcover types in our study area. Landcover types were obtained from the European Space Agency (ESA) based on Sentinel-1 and Sentinel-2 data at 10m resolution. Wetland had the highest NDVI values ($\bar{x}$= 45.63, SD = 11.2) followed by shrublands ($\bar{x}$= 18.58, SD = 8.9) and grasslands ($\bar{x}$= 14.25, SD = 6.6) and was lowest in barren/scarce vegetation ($\bar{x}$= 11.148, SD = 4.6).


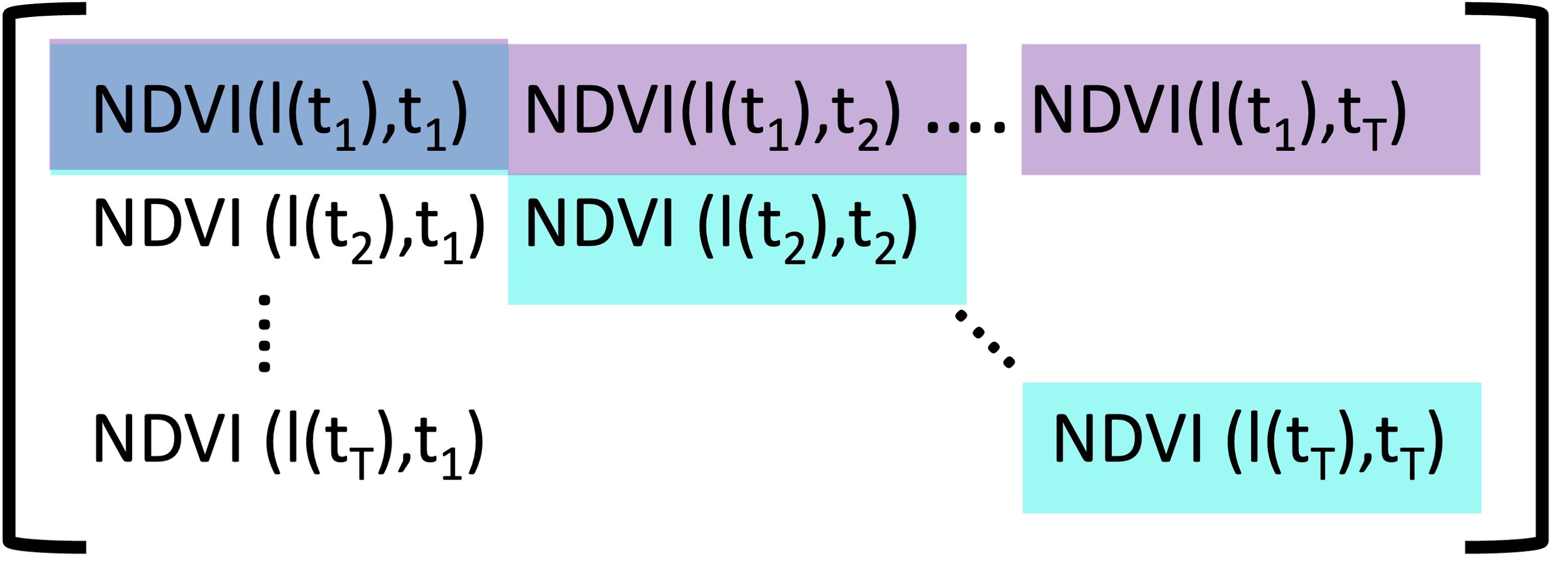


Figure 7. Schematic representation of the space-time-time matrix, where rows represent each location the individual used (l_i_); columns represent days (t_i_) that span throughout the duration of our study; and the diagonal (l(t_T_)(t_T_)) represents the day the animal was actually present at the corresponding location.

Table 1. Attributes of migration for 21 adult guanacos (Lama guanicoe) equipped with GPS collars in Patagonia, Argentina 2019-2022. Identification of seasonal behaviors (migratory, residential or dispersal) according to two methods, altitudinal Net Squared Displacement (NSD) and Seasonal Range Overlap, final classification and migration parameters regarding timing, duration and distance for individuals classified as migratory obtained from the top NSD model.

| ID | Starting range | NSD_Elev_ top model | NSD_Dist_ top model | Seasonal Overlap top model | Final classification | Summer departure date- θ | Winter departure date- θ | Duration- ρ (days) | Altitudinal Distance- δ (m) | Duration winter migration- φ (days) | Duration summer migration- φ (days) |
| --- | --- | --- | --- | --- | --- | --- | --- | --- | --- | --- | --- |
| G01 | Summer | Migratory | Migratory | Migratory | Migratory | 4/23/2019 | 9/21/2019 | 125 | 479 | 13 | 1 |
| G01 | Summer | Migratory | Migratory | Migratory | Migratory | 5/12/2020 | 10/10/2020 | 148 | 592 | 1 | 1 |
| G01 | Summer | Migratory | Migratory | Migratory | Migratory | 4/11/2021 | 9/1/2021 | 117 | 459 | 13 | 1 |
| G02 | Summer | Migratory | Migratory | Migratory | Migratory | 3/3/2019 | 10/7/2019 | 200 | 629 | 6 | 1 |
| G02 | Summer | Migratory | Migratory | Migratory | Migratory | 3/16/2020 | 10/26/2020 | 212 | 655 | 3 | 3 |
| G02 | Summer | Migratory | Migratory | Migratory | Migratory | 2/20/2021 | 10/27/2021 | 240 | 606 | 1 | 3 |
| G03 | Summer | Migratory | Migratory | Migratory | Migratory | 6/11/2020 | 8/11/2020 | 1 | 395 | 17 | 16 |
| G03 | Summer | Migratory | Migratory | Resident | Resident | NA | NA | NA | NA | NA | NA |
| G04 | Winter | Migratory | Migratory | Migratory | Migratory | 3/6/2020 | 10/6/2019 | 149 | 811 | 1 | 1 |
| G04 | Winter | Migratory | Migratory | Migratory | Migratory | 3/6/2021 | 11/3/2020 | 80 | 833 | 1 | 21 |
| G05 | Summer | Migratory | Migratory | Resident | Resident | NA | NA | NA | NA | NA | NA |
| G05 | Summer | Migratory | Migratory | Migratory | Migratory | 6/28/2020 | 8/11/2020 | 4 | 313 | 15 | 15 |
| G05 | Summer | Disperser | Migratory | Resident | Resident | NA | NA | NA | NA | NA | NA |
| G06 | Winter | Migratory | Resident | Disperser | Migratory | 1/8/2020 | 11/24/2019 | 34 | 369 | 1 | 21 |
| G07 | Summer | Migratory | Migratory | Migratory | Migratory | 4/22/2019 | 9/18/2019 | 96 | 521 | 17 | 4 |
| G07 | Summer | Migratory | Migratory | Migratory | Migratory | 3/20/2020 | 10/9/2020 | 134 | 623 | 21 | 12 |
| G07 | Summer | Migratory | Migratory | Migratory | Migratory | 3/24/2021 | 9/23/2021 | 193 | 455 | 1 | 1 |
| G09 | Summer | Migratory | Migratory | Migratory | Migratory | 7/3/2020 | 8/11/2020 | 1 | 295 | 11 | 4 |
| G09 | Summer | Migratory | Migratory | Resident | Resident | NA | NA | NA | NA | NA | NA |
| G10 | Winter | Migratory | Migratory | Disperser | Migratory | 9/23/2019 | 3/4/2019 | 101 | 576 | 1 | 2 |
| G11 | Summer | Migratory | Migratory | Migratory | Migratory | 2/8/2019 | 10/16/2019 | 211 | 475 | 10 | 7 |
| G11 | Summer | Migratory | Migratory | Resident | Migratory | 2/9/2020 | 10/20/2020 | 55 | 427 | 21 | 2 |
| G11 | Summer | Migratory | Disperser | Resident | Resident | NA | NA | NA | NA | NA | NA |
| G12 | Winter | Migratory | Migratory | Resident | Migratory | 2/25/2020 | 10/14/2019 | 89 | 510 | 21 | 1 |
| G13 | Winter | Migratory | Migratory | Resident | Resident | NA | NA | NA | NA | NA | NA |
| G13 | Winter | Resident | Resident | Resident | Resident | NA | NA | NA | NA | NA | NA |
| G14 | Winter | Migratory | Migratory | Disperser | Migratory | 3/20/2020 | 10/9/2019 | 140 | 667 | 10 | 1 |
| G15 | Summer | Migratory | Disperser | Disperser | Migratory | 3/17/2019 | 10/16/2019 | 130 | 775 | 21 | 21 |
| G16 | Winter | Migratory | Migratory | Disperser | Migratory | 2/9/2020 | 11/19/2019 | 21 | 1105 | 10 | 21 |
| G18 | Winter | Migratory | Migratory | Migratory | Migratory | 3/15/2021 | 10/10/2020 | 117 | 702 | 19 | 1 |
| G19 | Winter | Migratory | Migratory | Migratory | Migratory | 3/13/2021 | 10/25/2020 | 48 | 558 | 20 | 21 |
| G20 | Winter | Migratory | Migratory | Disperser | Migratory | 3/24/2021 | 10/20/2020 | 119 | 547 | 1 | 2 |
| G21 | Winter | Resident | Migratory | Resident | Resident | NA | NA | NA | NA | NA | NA |
| G24 | Summer | Migratory | Migratory | Migratory | Migratory | 5/21/2021 | 10/1/2021 | 97 | 579 | 17 | 5 |
| G26 | Summer | Migratory | Migratory | Resident | Resident | NA | NA | NA | NA | NA | NA |

Table 2. Intraindividual coefficient of variation for migratory guanacos with multiple years of data (3 individuals with 3 years of data and 2 individuals with 2 years of data) compared to intraindividual variation for all collared guanacos for different migratory characteristics.

|  | Intraindividual Coefficient of Variation (CV) | Interindividual coefficient of Variation (CV) |
| --- | --- | --- |
| Summer departure date | 11.2 | 51.2 |
| Winter departure date | 4.8 | 9.7 |
| Migration distance | 8.7 | 30.7 |
| Migration duration | 64.8 | 90.6 |
| Time spent in migratory range | 36.4 | 61.6 |


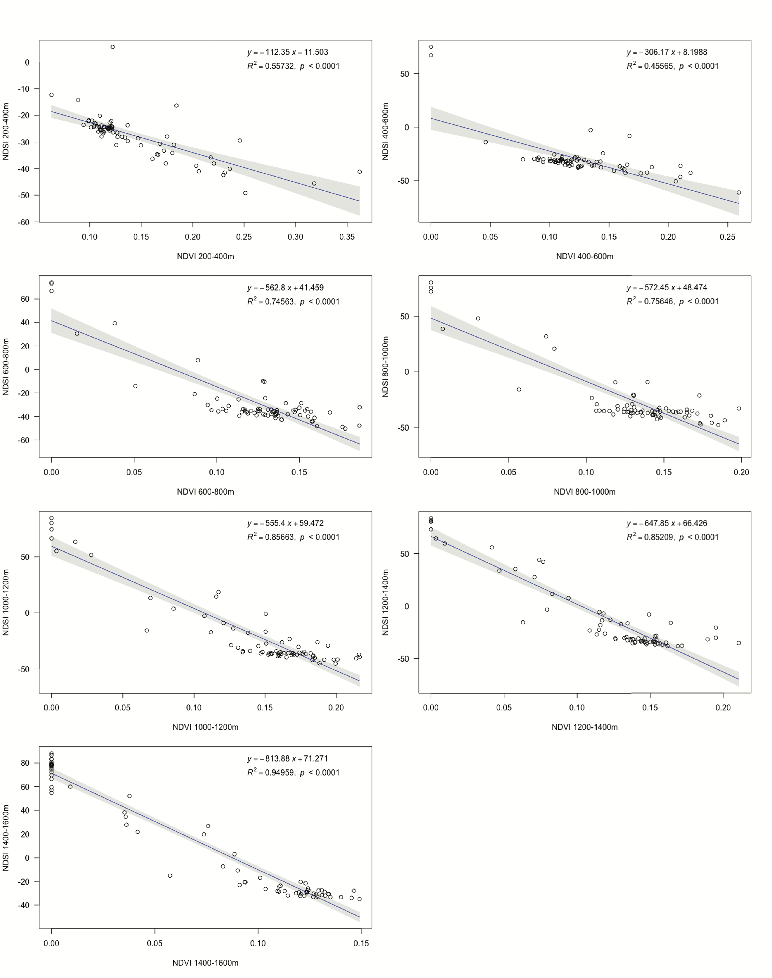


Figure 8. Plots of raw Normalized Difference Snow Index (NDSI) and Normalized Difference Vegetation Index (NDVI) summer 2019 to summer 2022, for 200 m altitudinal ranges covering the elevation gradient of the study area, in Patagonia, Argentina.
